# Switching Metazoan Fatty Acid Synthase Between Reducing and Nonreducing Elongation Mode via Programming of the Ketoreductase Domain

**DOI:** 10.1002/cbic.202500888

**Published:** 2026-01-29

**Authors:** Damian L. Ludig, André Herber, Martin Grininger

**Affiliations:** ^1^ Institute for Organic Chemistry and Chemical Biology Buchmann Institute for Molecular Life Sciences Goethe University Frankfurt Frankfurt am Main Germany

**Keywords:** enzyme catalysis, fatty acid synthase, ketoreductase, polyketides, protein engineering

## Abstract

Polyketides constitute a large class of natural products with important biological activities and applications such as antibiotics, antitumor agents, pesticides, and pigments. Their biosynthesis is catalyzed by polyketide synthases (PKSs) which are multidomain enzymes evolutionarily related to fatty acid synthases (FASs). Despite their close homology in structure and the chemistry they perform, FASs and PKSs differ fundamentally in their catalytic programming: FASs run fully reducing elongation reactions to yield saturated fatty acids, while iterative PKSs execute reductions just in selected cycles, generating complex oxidized compounds. In this study, we aimed at engineering the metazoan FAS in its ketoreduction (KR) domain to switch from fully reducing to a nonreducing mode during chain elongation. Guided by recent insights into KR programming, we incorporated a helix into metazoan FAS, which is found in KRs from iterative PKSs and type II FASs with chain length programming. These FAS variants initially catalyze complete fatty acid cycles but lose the ability of *β*‐keto reduction in later elongation rounds, producing intermediates that spontaneously cyclize to pyrone products. Finally, our study provides valuable insight into the mechanism of KR catalysis identifying another amino acid next to the active tyrosine which is capable for intermediate protonation.

## Graphical abstract

Striving to shed light on programming of iterative polyketide synthases (PKSs), we introduced a helix motif found in iterative PKS ketoreductase domains into an evolutionary related fatty acid synthase. With this, we were able to impose programming, switching between a reducing and nonreducing elongation mode. We ultimately enabled the production of acyl substituted pyrones and established a basis for bottom‐up programming of fatty acid synthases (FASs)/PKSs.

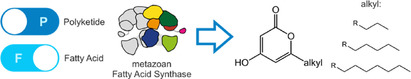

## 1 Introduction

**FIGURE 1:**
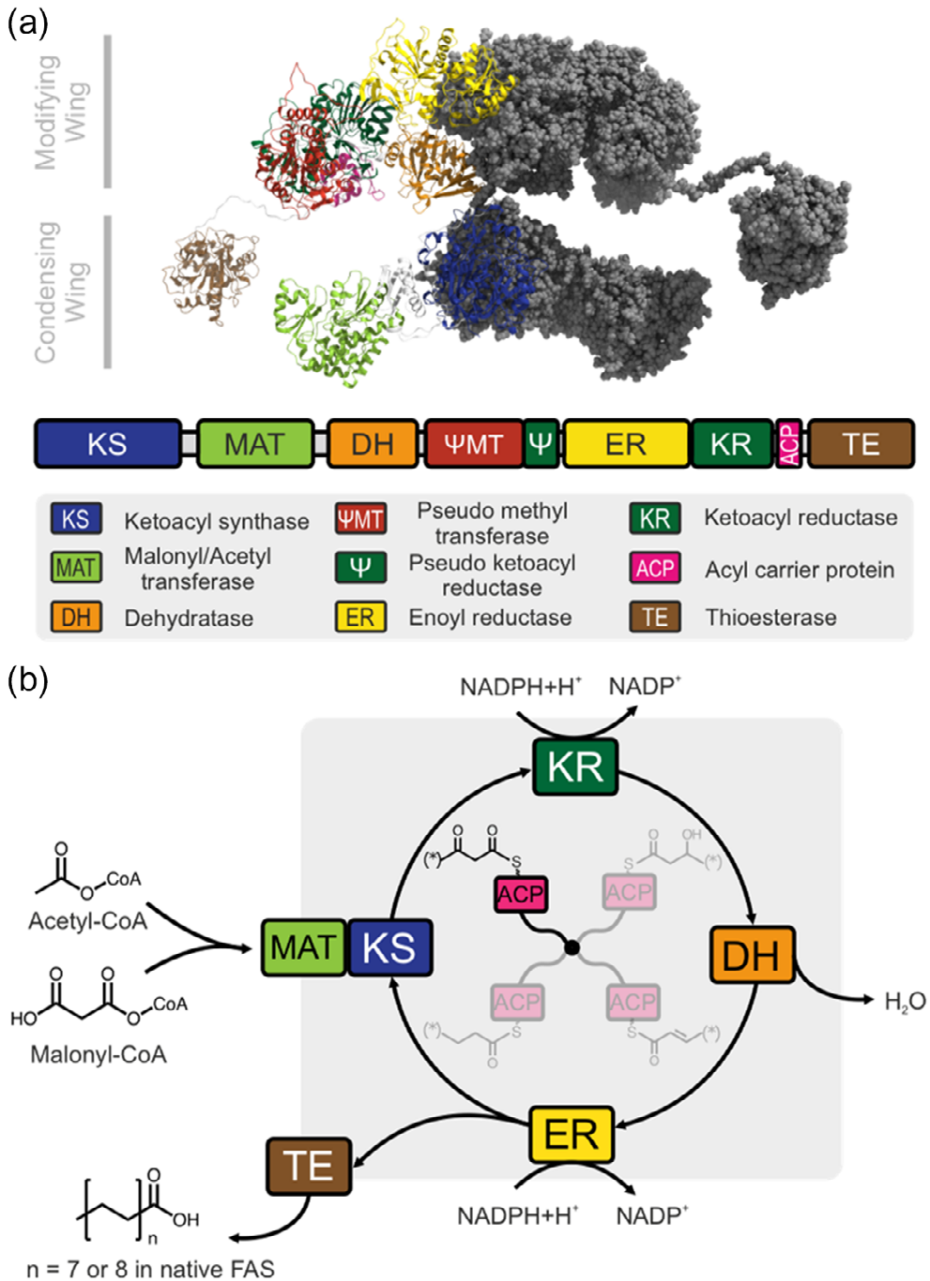
Fatty acid synthase and biosynthesis. (a) Depiction of the dimeric structure of mFAS [1]. The domains of one protomer are shown and color‐coded as given in the legend. Iterative PKSs share the overall architecture but modulate the modifying domain ensemble. (b) The fatty acid cycle performed by mFAS. During the whole synthesis, intermediates are either bound to a catalytic domain or the acyl carrier protein (ACP) which shuttles substrates and intermediates. The substrates acetyl‐ and malonyl‐CoA are loaded via the MAT. Then, KS forms a C—C bond in a Claisen condensation reaction, resulting in an intermediate which is elongated by two carbon atoms and bears a keto group at the *β*‐position (acetoacetyl‐ACP in cycle 1). This *β*‐position can subsequently be processed by three subsequent reactions: (i) reduction to the alcohol by the ketoreductase (KR) (hydroxybutyryl‐ACP), (ii) elimination of the hydroxy group by the dehydratase (DH) (crotonyl‐ACP), and (iii) reduction to a saturated chain by the enoylreductase (ER) (butyryl‐ACP). The resulting saturated acyl chain acts as substrate for the next elongation cycle. Acyl chain of chains length C16 or C18, received after seven or eight cycles respectively, are hydrolyzed from the ACP by the TE and released as free fatty acids.

Metazoan fatty acid synthase (mFAS), and iterative polyketide synthases (PKSs) are related enzymes that share a conserved multidomain organization and catalytic logic (Figure 1a,b) [2]. Two recent findings have greatly contributed to understanding their evolutionary relationships: First, the discovery of the animal FAS‐like polyketide synthases (AFPKs) bridged the phylogenetic gap between iterative PKSs and mFAS, linking secondary‐metabolite biosynthesis with primary lipid metabolism [3, 4]. Second, iterative type I PKSs were discovered to be widespread, not just in fungi but also in bacteria, linking iterative PKSs and bacterial modular PKSs through a shared evolutionary ancestry [5]. Thus, programming in iterative synthesis is not an isolated fungal innovation, but a deeply conserved catalytic strategy that provides insight into how complex polyketide structures have evolved [6].

PKSs are fascinating enzymes due to their ability to catalyze some of the most complex biosynthetic pathways in nature [7]. They are responsible for the biosynthesis of the large and diverse class of polyketides—secondary metabolites that function as antibiotics, antifungals, immunosuppressants, and other bioactive molecules. Beyond a fundamental biochemical interest in this type of proteins, research on PKSs has a large practical perspective, as their engineering offers to expand our therapeutic arsenal. Among PKSs, the modular type proteins are in focus of engineering approaches, because of their apparent programmability. In modular PKSs, each extension unit in the growing polyketide chain is processed by a dedicated module that is used only once during biosynthesis. These modules are arranged in sequence and perform an assembly‐line–like synthesis where the order of modules defines the structure of the final product. In contrast, iterative PKSs reuse a single module for multiple rounds of chain extension, making them far less susceptible to engineering. However, the strength in iterative PKSs lies in providing an enzyme‐economical yet chemically highly sophisticated biosynthesis. Understanding how these systems control the sequence and outcome of catalytic events, remains one of the central challenges to their rational engineering [8].

FASs have served as tractable models for engineering lipid biosynthesis [9, 10, 11]. Their catalytic working mode is strictly uniform to yield saturated products. Considering the decision‐making logic of the evolutionarily iterative PKSs, we asked whether the fatty acid biosynthetic regime could be relaxed and mFASs reprogrammed to perform context‐dependent catalytic cycles reminiscent of polyketide assembly. Imposing this type of reaction programming onto mFAS would be highly rewarding from an engineering perspective, as mFAS turned out to be a fast [12] and engineerable protein [13, 14].

**FIGURE 2:**
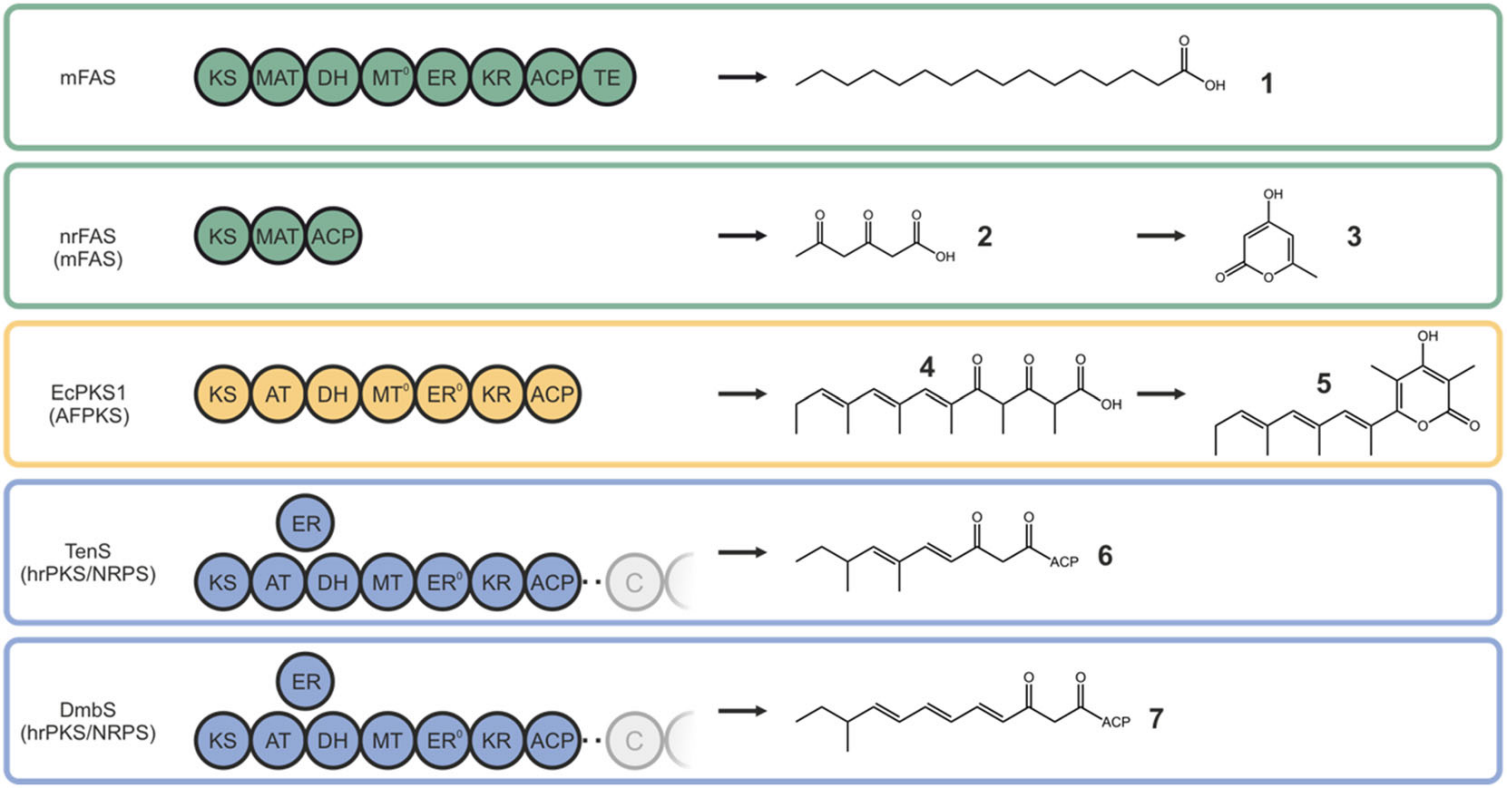
Related FAS/PKS multienzymes and their corresponding products. Domain orders of representatives of mFASs (green), AFPKs (yellow), and hrPKS/NRPS (blue and only PKS part shown). For each protein, the corresponding product or ACP‐tethered intermediate is shown. mFAS produces palmitic acid (**1**), nrFAS and EcPKS1 produce triketides (**2** and **4**) which form pyrones (**3** and **5**) and TenS and DmbS form ACP‐tethered diketides (**6** and **7**).

Here, we specifically focus on the ketoreductase (KR) domain, which emerges as a particularly compelling target for programmed iterative synthesis. The KR is a key domain in the programming of the AFPK from *E. chlorotica* (EcPKS1) [3], the tenellin hrPKS/NRPS (TenS) [15] or the desmethylbassianin hrPKS/NRPS (DmbS) (Figure 2) [16]. The products of EcPKS1, TenS and DmbS feature reduced regions at the *ω*‐end of the chain, while retaining unreduced keto‐group(s) in the *β* (and δ) positions. This observation suggests the presence of a regulatory switch that turns off ketoreduction after initial reductive elongations. As a result, the products appear as arising from the sequential action of a (fully) reducing FAS‐like synthesis and nonreducing PKS‐like synthesis, which can also be achieved by a nonreducing FAS (nrFAS) lacking the KR, DH, ER, MT^0^ and TE domain (Figure 2) [17]. In the case of TenS and DmbS, this switch seems to be chain length dependent. Yang et al*.* discovered a helix near the catalytic center of the KR that is responsible for the chain length differences in the TenS/DmbS systems and appears to play a major role in programming of the KR domain [18].

The pyrone scaffold seen in products **3** and **5** can be found in several natural products with diverse biological activities. For example, 6‐alkyl pyrones such as fistupyrone show antifungal activity [19], while different 2,6‐substituted pyrones show antibiotic, antiviral, antiparasitic, and anticancer properties [20, 21, 22].

Building on insights from different KR domains, we aimed at reprogramming the KR of mFAS to modulate its reductive behavior. We show that, depending on the specific modification of the KR domain, we can switch between reductive FAS‐like elongation and nonreductive PKS‐like elongation at different chain lengths, enabling the production of pyrones with a saturated acyl chain moiety. Moreover, this work provides valuable insights into the mechanism of the KR and the roles of the catalytically active amino acid residues.

## 2 Results and Discussion

### 2.1 Structural Evaluation of KR Programming

**FIGURE 3:**
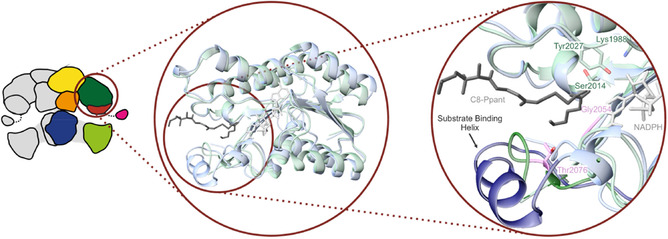
Comparison of mFAS KR and TenS KR. Superposition of the human FAS KR (PDB ID: 8EYI) [23] with an Alphafold 3 model of the KR from TenS. Highlighted are the catalytic triad of mFAS KR in green, two positions for point mutations performed in this study in rose, the substrate binding helix of TenS in dark blue and the region which was replaced by the helix in mFAS KR in dark green. The NADPH molecule (light gray) is part of the cryo‐EM structure of human FAS KR, the C8‐phosphopantetheine (dark gray) is positioned from an overlay with *H. pylori* FabG (PDB ID: 8JFG, Figure S1) [24].

To gain insights into the structural determinants of KR programming, we compared the structures of six different KRs (mFAS, EcPKS1, TenS, DmbS, and FabG from *H. pylori* and *E. coli*) (Figure 3 and Figure S1). The structural alignment of the domains showed their similarity in the overall fold and in the active site residues. A major difference was the presence of the substrate binding helix in TenS and DmbS at a position where mFAS contains a flexible loop. Interestingly, FabG features a similar helix to TenS. Specificity data for the *H. pylori* FabG indicate a strong preference for shorter acyl chains [24], implying a similar way of KR regulation despite its origin from a type II FAS system. In contrast, EcPKS1 lacks the helix structural motif, indicating that the presence of this feature is not the only means of achieving this programming. In our efforts to program mFAS KR, we pursued two different strategies. First, we restricted the space around the catalytic center, next to the active Serine/Tyrosine and the NADPH hydride, by installing sterically demanding amino acids. Second, we replaced the flexible loop with the substrate binding helices from TenS, DmbS, and FabG from *E. coli* and *H. pylori*. (Figure 3).

### 2.2 Pyrone Formation Activity Assay

mFAS is able to form the pyrone triacetic acid lactone (TAL), when no NADPH is present [25] or as side product in small quantities during standard mFAS synthesis [26]. As observed for EcPKS1 [27] or nrFAS [17], the formation of a pyrone is possible without involving an TE domain, which suggests that the triketide, covalently bound to the ACP, releases spontaneously upon pyrone formation (Figure 2 and Figure S2) [28].

We harnessed this reaction mode to establish an assay in which an mFAS lacking the TE domain, termed mFASΔTE, is modified in the KR domain. With a KR that has active chain length control, mFASΔTE would first enable reductive elongations to receive a fully saturated acyl chain bound to the ACP. At a certain chain length, the KR would then halt reductions and induce nonreductive elongations to the triketide that, once formed, releases spontaneously as pyrone [29]. The pyrone formation can be monitored photometrically, as the absorption at 285 nm is directly proportional to the amount of pyrones formed. We used mFASΔTE as negative control, since it should not produce pyrones in significant amounts under the assay conditions, and nrFAS only consisting of KS, MAT, and ACP, producing TAL, as positive control [17]. We further constructed two different KR knockouts following the design for 6‐deoxyerythronolide B synthase KR6 reported earlier (S2014A and Y2027F, mouse FAS numbering) [30]. These residues stabilize the acyl‐substrate and mediate the proton transfer after the nucleophilic attack of the hydride (Figure S3) [31, 32, 33].

**FIGURE 4:**
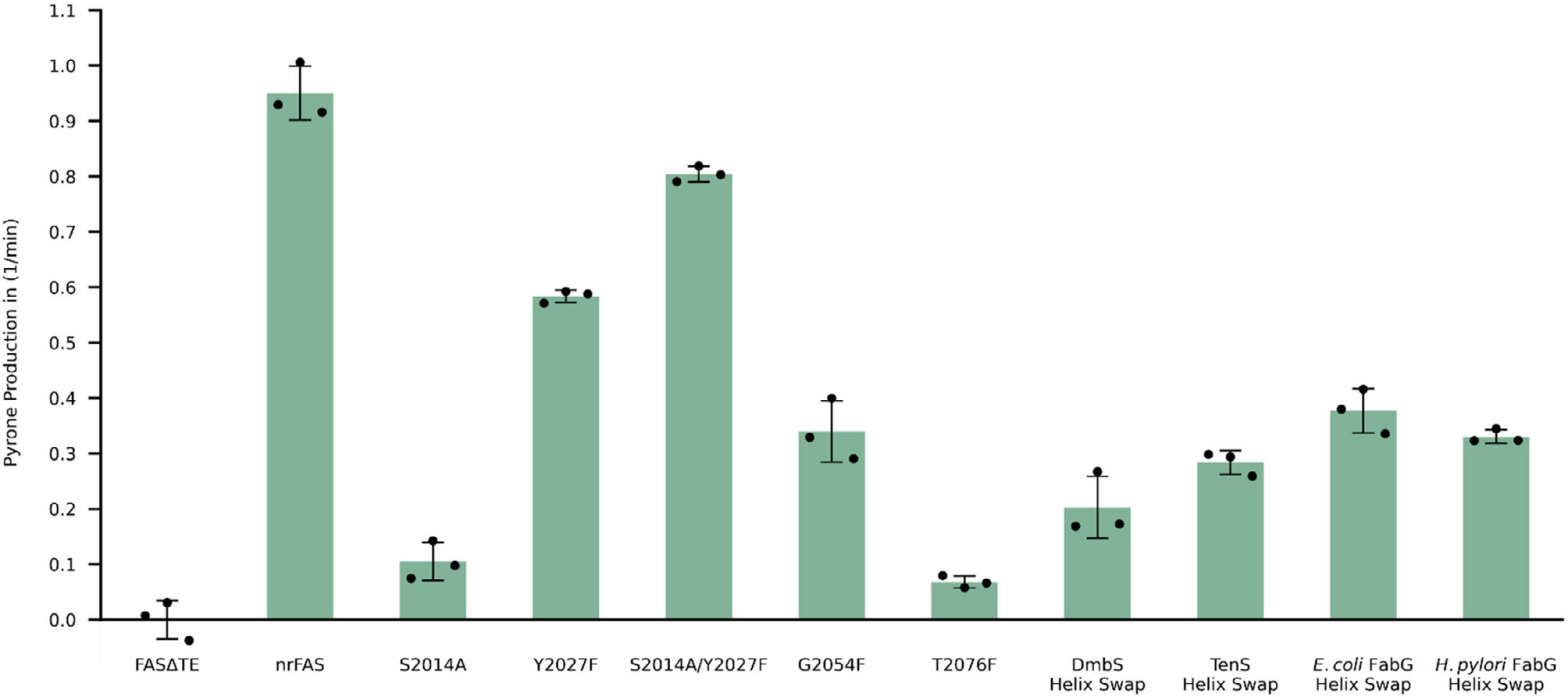
Pyrone formation activity of the KR mutant variants. Pyrone production rate of mFAS mutants monitored by the absorption at 285 nm. Data were collected in biological triplicates, each with three technical replicates. Individual biological replicates are shown as datapoints, the bars represent the means and error bars refer to the standard deviation. mFASΔTE was used as blank in this experiment.

The pyrone formation assays revealed large differences in the effectiveness of our approaches (Figure 4). Variants of mFASΔTE, harboring knockout mutations S2014A, Y2027F, and S2014A/Y2027F, produced pyrones at different rates. The single point mutations in KR, designed to create steric hindrance, performed differently. While G2054F showed some pyrone production, T2076F apparently failed to disrupt the reductive fatty acid cycle. Finally, most promising results were obtained for the constructs in which the substrate binding helices were inserted. Notably, all four of these constructs were produced in good purity, which is remarkable considering that the inserted substrate binding helices differ between 75% and 94% from the mFAS sequence (Figure S4–S6). All these constructs displayed pyrone production at slightly differing rates. Three helix constructs exhibited about a third of the nrFAS's activity in pyrone formation, with the constructs with helices from FabG showing slightly higher activity compared to the TenS helix construct. In comparison, the DmbS construct shows the slowest rates with about a quarter of the activity of nrFAS.

### 2.3 Product Assay to Determine Chain Length Profile

Building on the outcomes from the pyrone formation activity assay, we next analyzed the constructs regarding the specific pyrones generated. These assays were performed to obtain a fingerprint of each construct's ability for reductive elongations prior to pyrone formation. Depending on the type of KR modification, we expected different outcomes: (i) KR knockout variants of mFASΔTE were expected to completely prevent reduction, ultimately leading to the production of TAL [25]. (ii) Binding site mutants in which small amino acids were replaced by bulkier residues were expected to overall hamper KR activity, leading to an overall broad distribution of pyrones at low concentrations. (iii) Helix‐insertion variants were expected to most effectively realize the chain length dependent switch, enabling the production of pyrones with an acyl chain.

**FIGURE 5:**
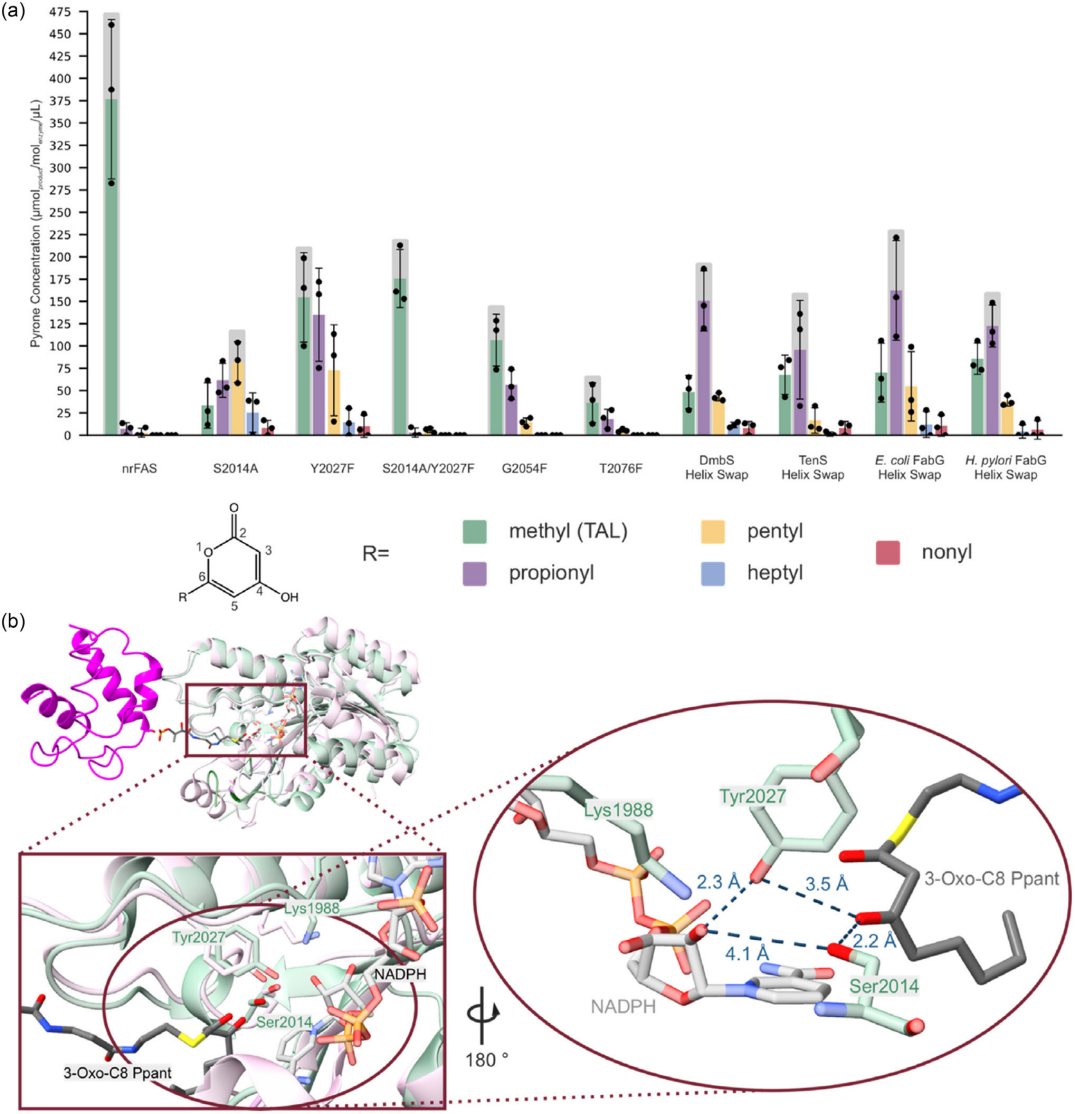
Production of pyrones with different acyl chains on position 6. (a) Product profiles of mFAS mutants analyzed by LC‐MS measurements. The major product is highlighted by a gray box. Data were acquired from three independent biological replicates, each measured as three technical replicates. Dots represent individual biological replicates, the bars represent the means and error bars indicate the standard deviation. A representative LC‐MS analysis can be found in Figure S7. (b) Overlay of the active sites and position of the ACP‐bound 3‐Oxo substrate in the active site. A further zoom shows the distances of the catalytic serine and tyrosine to the keto‐oxygen and the ribose hydroxygroup, which are the two direct partners in the proton transfer during the reduction. mFAS and NADPH (PDB ID: 8EYI) [23], *E. coli* FabG and ACP‐bound substrate (PDB ID: 1Q7B) [31].

The binding site mutants (G2054F and T2076F) showed overall poor switching properties, producing only minor amounts of pyrones with up to two reductive elongations and TAL as clear main product (Figure 5a). This indicates that KR activity is decreased to a level such that KS generally outcompetes KR in processing the *β*‐carbonyl intermediate (acetoacetyl‐ACP, Figure 1b). In contrast, for the substrate helix constructs, we detected pyrones resulting from up to four reducing elongations before formation of the triketide, although all have propionyl‐pyrone as main product. Thus, these variants usually perform one reductive cycle before switching to unreduced elongation. The knockout mutants produced unexpected results, as both the S2014A and Y2027F mutants retained reductive elongation activity. Only when both active residues were mutated simultaneously, the KR was inactive and mFASΔTE exclusively produced TAL. Our data show that the two residues do not contribute to catalysis equally, as mutation Y2027F led to fewer reductive elongations than S2014A. This indicates that Y2027 plays the more critical catalytic role, consistent with its established function as the residue responsible for the protonation of the reaction intermediate (Figure S3) [32, 33, 34]. However, in absence of Y2027, S2014 seems capable of partial compensation, likely by mediating proton transfer through a suitable positioning in the active site (Figure 5b). The main role of S2014 appears to lie in aligning the substrate within the active site, a function that Y2027 still can partially maintain in the S2014A mutant.

Overall, the substrate helix swap constructs displayed a narrower product distribution of pyrones compared to the knockout mutants. This is reflected by higher relative abundances of the predominant pyrone chain length over all detected pyrones: 0.39 and 0.40 for S2014A and Y2027F, respectively and 0.58 (DmbS), 0.51 (TenS), 0.52 (*E. coli* FabG), and 0.48 (*H. pylori* FabG). These results demonstrate that the substrate helix adds a layer of substrate specificity onto the mFAS KR, enabling the switch of mFAS from reducing to nonreducing synthesis at defined chain acyl length. We propose that, at a certain chain length, the *β*‐ketoacyl intermediate tethered to the ACP becomes sterically hindered in its interaction with the KR due to the presence of the substrate‐binding helix and is then steered towards KS‐mediated elongation of the unreduced intermediate.

The broader product spectra for mutants S2014A and Y2027F suggest that product output is governed by the kinetic interplay between the KS and a KR whose catalytic efficiency is generally compromised by the knockout mutations, independent of chain length. Particularly, in the KR variant S2014A, the reduced efficiency of KR appears to closely match the KS efficiency for *β*‐keto intermediates. The data suggest that KR dominates over KS for the shorter chain intermediates, while the C8 intermediate (*β*‐keto‐octanoyl‐ACP) is then preferentially processed by the KS. In contrast to the S2014A and Y2027F mutations, insertion of a helix into the KR appears to induce a chain length specific decrease in KR activity, resulting in faster reduction of shorter chains and a pronounced decline for longer chains, thereby leading to sharper product spectra.

Thus, the relative catalytic efficiencies of the KR and KS for *β*‐ketoacyl chains at a given chain length determine product output. Likewise, the kinetic interplay of the KS and the TE domains has recently enabled fatty acid chain length control in mFAS [13, 35].

We note that a direct correlation between the overall product formation and the activity assays is not possible due to the necessity of differing assay conditions (e.g., NADPH concentration and reaction time). No products other than the pyrones were detected.

## 3 Conclusion

In this work, we investigated the possibilities of switching mFAS between reducing fatty acid and nonreducing polyketide synthesis through the engineering of the KR domain. Via introduction of a substrate binding helix found in some PKS and type II FAS KRs, we achieved the programming of the mFAS KR. In the engineered mFAS, the chain length dependent catalytic efficiencies of the modified KR and KS governed the transition from reductive to nonreductive processing. Specifically, induced by the substrate helix, the KR became kinetically outcompeted by the KS, such that after initial reducing elongation(s), elongation proceeded without reduction, ultimately yielding the triketide that is released as a pyrone. This engineering led, to the best of our knowledge, to the first programmed mFAS KR. The resulting pyrones can serve as direct precursors for the synthesis of more elaborate 3,6‐substituted pyrones, an interesting class of natural products like the antifungal Fistupyrone or the antitumor agent Violapyrone C [36].

In this work, we demonstrate that chain length programming exhibited by the KR domain in certain iterative PKSs is a transferable feature that can be imposed onto the mFAS framework. This adds an additional layer to the toolbox of mFAS programming and highlights mFAS as a highly engineerable scaffold for programmed biosynthesis [12, 13]. From a broader perspective, our results provide evidence that the catalytic decision‐making in iterative polyketide synthesis, also referred to as cryptic programming, is modular and transferable, rather than emerging as a complex system‐level property. Our findings, therefore, establish a conceptual foundation for the bottom‐up programming of biosynthetic pathways within iterative PKSs or in FASs.

## 4 Experimental Section

### 4.1 Plasmid Transformation

1 μL of the chosen plasmid was added to 50 μL of electrocompetent BAP 1 cell suspension. This mixture was chilled on ice before its transfer to an equally cooled 0.2 cm electroporation cuvette and an electric shock was applied (25 μF, 200 Ω, 2.5 kV). The suspension was immediately removed with the aid of 300 μL prewarmed SOC medium (37°C) and incubated at 37°C for 1 h. The cell suspension was centrifuged at 600 rcf for 1.5 min before removal of 300 μL of the supernatant. The cell pellet was resuspended in the remaining supernatant and spread on LB‐Agar plates containing 100 $\frac{\text{µ}g}{\mathrm{mL}}$ ampicillin (Amp) before incubation at 37°C overnight.

### 4.2 Gene Expression and Protein Purification

20 mL of LB medium containing 100 $\frac{\text{µ}g}{\mathrm{mL}}$ Amp per 1 L of main culture were inoculated with five colonies and incubated overnight at 37°C and 140 rpm. The chosen volume of TB medium containing 100 $\frac{\text{µ}g}{\mathrm{mL}}$ Amp was then inoculated with the preculture and incubated at 37°C and 140 rpm until an optical density (OD) of 0.6–0.8 was reached. The flasks were cooled at 4°C for 30 min before protein expression was induced by adding 250 μL of 1 M isopropyl *β*‐d‐1thiogalactopyranoside (IPTG) per liter main culture. The expression was carried out at 20°C and 140 rpm overnight. The cells were harvested by centrifugation using an Avanti J20‐XP centrifuge with a JLA 8.100 rotor (Beckman Coulter) at 5000 rcf and 4°C for 20 min. The supernatant was discarded, and the cell pellet was resuspended in 25 mL Ni‐Wash buffer containing a catalytic amount of DNase I and 70 μL of 0.5 M EDTA.

Disruption of the cells was performed at 800 bars using an Amico French Pressure Cell Press (SLM Aminco). The resulting suspension was centrifuged at 50 000 rcf and 4°C for 1 h using an Avanti J20‐XP centrifuge with a JA 25.500 rotor (Beckman Coulter). The supernatant was transferred into a fresh 50 mL falcon tube containing 70 μL of 1 M MgCl_2_ solution. The crude extract was further purified by affinity chromatography.

The protein of interest was purified from the crude extract by nickel affinity chromatography (3 mL column volume, CV) and following Strep‐Tactin XT chromatography (2 mL CV). Both affinity chromatographic methods include wash and elution phases, each performed with 5 CV of the appropriate buffer (Ni‐Wash, Ni‐Elution, Strep‐Wash, or Strep‐Elution, as applicable). The final elution was concentrated to a volume of approximately 1 mL using an Amicon Ultra centrifugal filter unit (Merck). The molecular weight cutoff was chosen appropriately to the protein size. The concentrated protein was stored at −70°C. All affinity chromatography steps were monitored via SDS‐PAGE analysis.

Size exclusion chromatography (SEC) was performed at 4°C with an ÄKTA Explorer (Cytivia) or an ÄKTA Purifier (Cytivia) equipped with a Superdex 200 Increase 10/300 GL column (Cytiva). All samples were filtered using a 0.22 μm Ultrafree centrifugal filter (Merck) before application. mFAS samples were incubated at 37°C for 1 h prior to centrifugation to promote dimerization. Fractions were selected based on the absorbance measured at 280 nm. The combined fractions were concentrated as desired using an Amicon Ultra centrifugal filter unit (Merck) with the appropriate properties. Aliquots were stored at −70°C.

### 4.3 Absorption‐Based Activity Assay

The activity assay was performed in mFAS assay buffer, the dithiothreitol (DTT) and bovine serum albumin (BSA; final concentration of 0.03 $\frac{\text{µ}g}{\mathrm{mL}})$ added on the day of the assay. Samples were prepared in a 384‐cavity reaction plate (Greiner Bio‐One) with a sample volume of 100 μL and measured with a CLARIOstar Plus plate reader (BMG Labtech) at 25°C. Further settings were set to 240 cycles, 22 flashes per well and cycle, and 15 s cycle time coming to a measurement time of 1 h in which the absorption at 285 nm was monitored. The final concentrations were: 200 µM NADPH, 200 µM Acetyl‐CoA, 600 µM Malonyl‐CoA, and 2 µM enzyme. All enzymes analyzed were purified via SEC. Blank measurements did not include any CoA‐esters and were measured using the nrFAS. Samples were prepared in technical triplicates. A calibration curve prepared with TAL was used for all constructs.

### 4.4 Product Quantification by Liquid Chromatography‐Mass Spectrometry

The product assay was performed in mFAS assay buffer, the DTT and bovine serum albumin (BSA; final concentration of 0.06 $\frac{\text{µ}g}{\mathrm{mL}})$ added on the day of the assay. The reaction mixtures with a volume of 150 µL were prepared in 1.5 mL Eppendorf tubes. The final concentrations were: 1400 µM NADPH, 133 µM Acetyl‐CoA, 400 µM Malonyl‐CoA, and 2 µM enzyme. All enzymes analyzed were only purified via affinity chromatographic methods. Samples were kept at room temperature in the dark overnight. Subsequently, 15 μL of 0.2 $\frac{g}{L}$ aqueous olivetol was added as the internal standard. 100 μL acetate buffer was added for improved phase separation. The extraction was performed three times with 200 μL ethyl acetate (≥99.7%, Honeywell). The solvent was removed in a SpeedVac Concentrator SVC‐100H (Savant) and substituted with 50 μL methanol (HPLC‐grade). After 20 min of centrifugation at 20 000 rcf, 40 μL were submitted in an HPLC‐vial for liquid chromatography‐mass spectrometry (LC‐MS) measurement. Samples were measured on HPLC‐ESI‐MS using the Ultimate 3000 LC (Dionex) system equipped with an Acquity UPLC BEH C18 (2.1 × 50 mm, particle size 1.7 µm, Waters) and connected to an AmaZonX (Bruker).

Data was analyzed using msconvert [37] and MZmine [38]. The TAL derivatives were confirmed by searching for the theoretical m/z for the [M + H]^+^‐ion. The integrals were further statistically processed using a custom python script using Pandas [39] and Matplotlib [40].

The signal‐to‐noise ratio in the extracted ion chromatograms for the analyzed heptyl‐ and nonyl‐pyrone species reached a low value for most samples. Despite this, these species remained as analytes as individual samples showed their elevated abundance.

## Supporting Information

Additional supporting information can be found online in the Supporting Information section. **Supporting Fig. S1: Structures of six KRs from different systems and their overlayed binding regions.** mFAS (PDB ID: 8EYI)^[14]^, TenS (Alphafold 3), H. pylori FabG (PDB ID: 8JFG)^[13]^, EcPKS (PDB ID: 9QC9)^[27]^, DmbS (Alphafold 3), *E. coli* FabG (PDB ID: 1Q7B)^[21]^. **Supporting Fig. S2:** **Mechanism of the pyrone formation through lactonization of a triketide.** A triketide is released from the ACP by cyclization, followed by a keto‐enol tautomerization reaction to leading to a TAL derivative. R represents a variable residue, which, in the case of TAL, is a methyl group. **Supporting Fig. S3:** **Putative mechanism of the KR domain.** Ser2014 and Tyr2027 position the β keto group for the nucleophilic attack from the hydride of NADPH. When attacked by the hydride, carbonyl oxygen gets protonated by the hydrogen of Tyr2027. Subsequently Tyr2027 gets reprotonated from Lys1988 via proton relay over a hydroxy group of the ribose ring. **Supporting Fig. S4: Sequence alignment and similarity matrix of the substrate binding region.** Aligned is the region which builds the substrate binding helices in TenS, DmbS and FabG as well as their counterparts in EcPKS and mFAS. The Alignment was created with Unipro UGENE using the Clustl Algorithm. **Supporting Fig. S5:** **Representative SDS‐PAGE analysis of the protein purification procedure.** The evident molecular weights of mFAS mutants recombinantly produced in *E.* *coli* align with the calculated molecular weights of 243–244 and 108 kDa of the mFASΔTE and the nrFAS respectively. **Supporting Fig. S6:** **Size** **exclusion** **chromatogram of all constructs.** The shown normalized chromatograms are representative of all mFAS mutants produced. All mFASΔTE variants show a pronounced peak at 10–11 mL elution volume corresponding to the dimeric state of the construct. A following shoulder indicates remaining monomeric protein. A preceding peak/shoulder at 9 mL elution volume points to aggregate. The chromatogram of the nrFAS exhibits the analogues characteristics with a shift +2 mL elution volume. Fractions of the dimeric state were concentrated and used for further experiments. **Supporting Fig. S7:** **Representative extracted ion** **chromatograms of the via LC‐MS analyzed product species.** The chromatograms depicted originate from one technical sample of the mFASΔTE *E.* *coli* FabG Helix Swap mutant. Extracted are the chromatograms for the protonated species of the expected products with an *m*/*z* tolerance of 0.03 (TAL: 127.03, propyl‐pyrone: 155.07, pentyl‐pyrone: 183.10, heptyl‐pyrone: 211.13, nonyl‐pyrone: 239.16). Peaks targeted for the automated integration are highlighted using gray boxes. **Supporting Table S1:** Buffers used in this work. **Supporting** **Table S2:** Media used in this work. **Supporting Table S3: Primers used in this work.** The fragments for the In‐Fusion Cloning containing mutations in the mFAS KR were created using the following primers. Template for all constructs was pAR88 (mFASΔTE). **Supporting Table S4: Sequence information of the constructs used in this work.** The amino acid and DNA sequences of the constructs pAR88 (mFASΔTE) and pAR127 (nrFAS), as well as for the swapped helices of DmbS, TenS, the *E. coli* FabG, and the *H. pylori* FabG are listed below. The mutated positions are highlighted.

## Conflicts of Interest

The authors declare no conflicts of interest.

## Supporting information

Supplementary Material
