# Supplementary material for: Switching Metazoan Fatty Acid Synthase Between Reducing and Nonreducing Elongation Mode via Programming of the Ketoreductase Domain
Source: Chembiochem. 2026 Jan 29;27(2):e202500888. doi: 10.1002/cbic.202500888 (PMC12856111; doi:10.1002/cbic.202500888)
Supplement: Supplementary file 1 — Supplementary Material [file CBIC-27-e202500888-s001.pdf]

Supporting Information

Switching Metazoan Fatty Acid Synthase Between Reducing and Non-reducing Elongation Mode via Programming of the Ketoreductase Domain

Damian L. Ludig[1,+,*], André Herber[1,+], Martin Grininger[1,*]

[1] *Institute of Organic Chemistry and Chemical Biology, Buchmann Institute for Molecular Life Sciences, Goethe Universität Frankfurt am Main Max-von-Laue-Str. 15, 60438 Frankfurt, Germany*

+ *These authors contributed equally.*
* *Corresponding author: grininger@chemie.uni-frankfurt.de, ludig@chemie.uni-frankfurt.de*

Buffers and Media

Table S1: Buffers used in this work.

Name	Composition	pH
mFAS assay buffer	50 mM KPi, 10% glycerol, 5% PEG 400, 1 mM DTT	7.0
Ni-Elution	200 mM KCl, 300 mM imidazole, 50 mM K_3PO_4, 10% glycerol	7.0
Ni-Wash	200 mM KCl, 30 mM imidazole, 50 mM K_3PO_4, 10% glycerol	7.0
Strep-Elution	250 mM K_3PO_4, 1 mM EDTA, 10% glycerol, 50 mM biotin	7.0
Strep-Wash	250 mM K_3PO_4, 1 mM EDTA, 10% glycerol	7.0
acetate buffer	95.2 mM acetic acid, 4.83 mM sodium acetate	

Table S2: Media used in this work.

Name	Composition	pH
LB Agar (Carl Roth)	1.2% (w/v) bacto agar, 1% (w/v) tryptone, 0.5% (w/v) yeast extract, 0.5% (w/v) NaCl	7.0
LB medium (Carl Roth)	1% (w/v) tryptone, 0.5% (w/v) yeast extract, 0.5% (w/v) NaCl	7.0
SOC medium	2% tryptone, 0.5% yeast extract, 10 mM NaCl, 2.5 mM KCl, 10 mM MgCl2, 10 mM MgSO4, and 20 mM glucose	-
TB medium	1.2% (w/v) tryptone, 2.4% (w/v) yeast extract, 0.5% (v/v) glycerol, 100 mM phosphate buffer pH 7.5	7.5

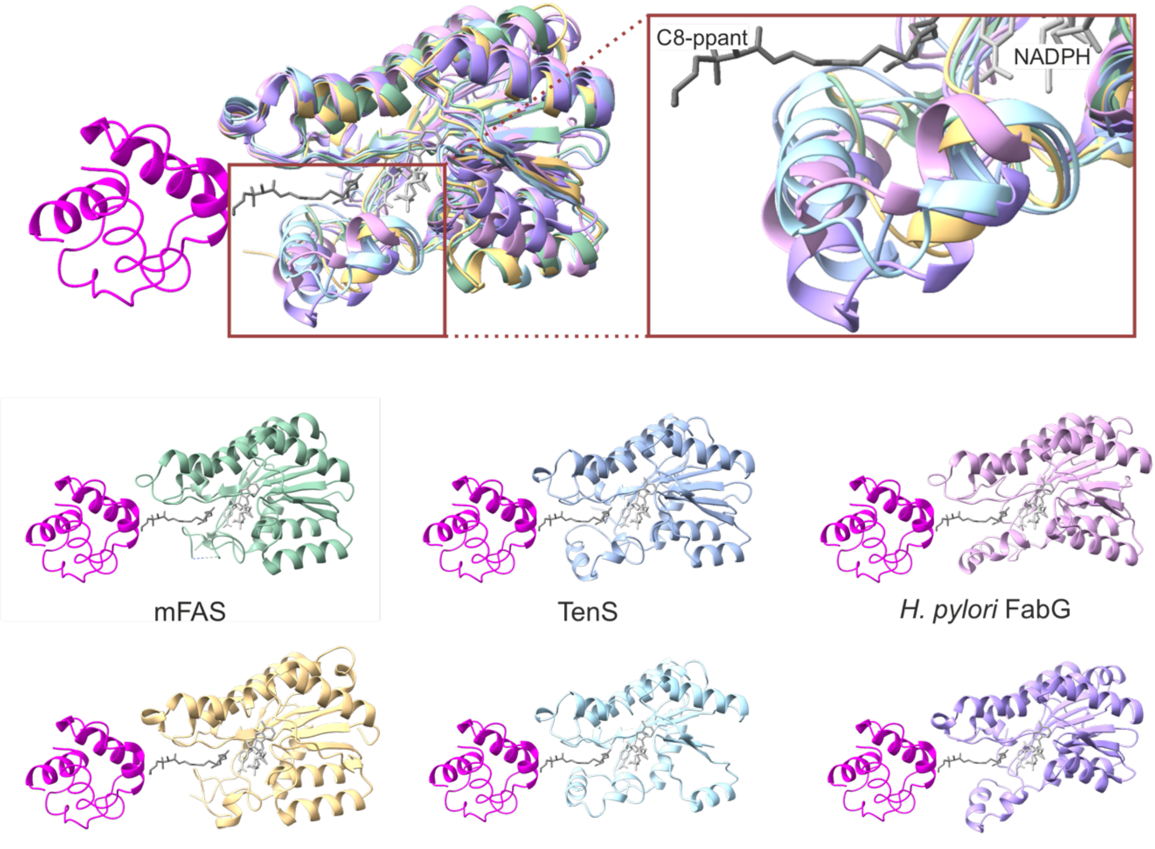

Figure S1 | Structures of six KRs from different systems and their overlayed binding regions. mFAS (PDB ID: 8EYI)[14], TenS (Alphafold 3), H. pylori FabG (PDB ID: 8JFG)[13], EcPKS (PDB ID: 9QC9)[27], DmbS (Alphafold 3), E. coli FabG (PDB ID: 1Q7B)[21].

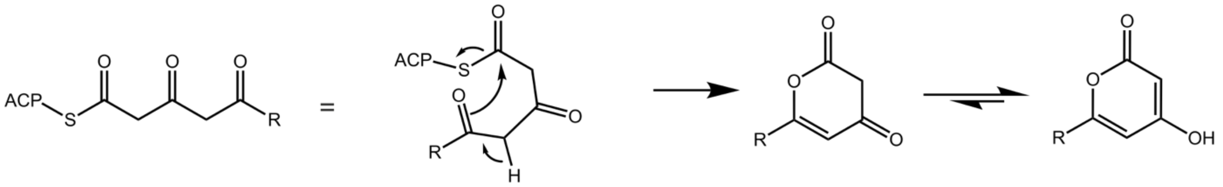

Figure S2 | Mechanism of the pyrone formation through lactonization of a triketide. A triketide is released from the ACP by cyclization, followed by a keto-enol tautomerization reaction to leading to a TAL derivative. R represents a variable residue, which, in the case of TAL, is a methyl group.

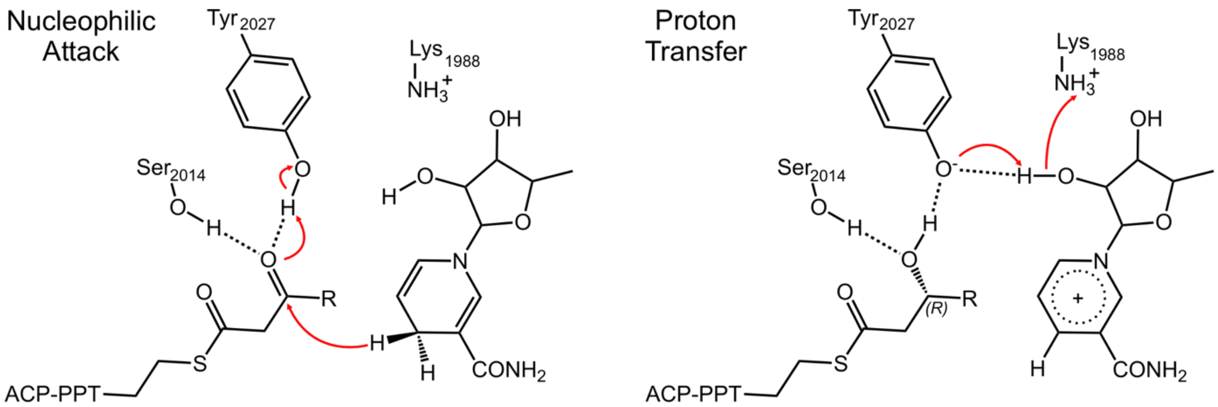

Figure S3 | Putative mechanism of the KR domain. Ser2014 and Tyr2027 position the β-keto group for the nucleophilic attack from the hydride of NADPH. When attacked by the hydride, carbonyl oxygen gets protonated by the hydrogen of Tyr2027. Subsequently Tyr2027 gets reprotonated from Lys1988 via proton relay over a hydroxy group of the ribose ring.

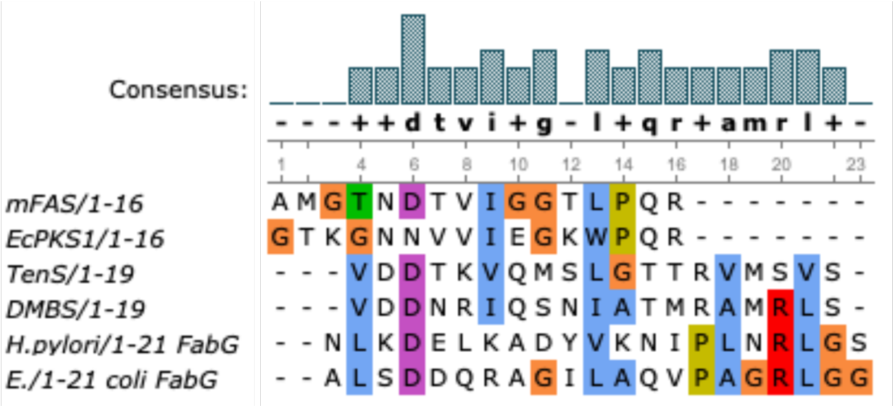

	mFAS/1-16	EcPKS1/1-16	TenS/1-19	DMBS/1-19	H.pylori/1-21 FabG	E./1-21 coli FabG
mFAS/1-16	100%	44%	19%	13%	6%	25%
EcPKS1/1-16	44%	100%	0%	6%	0%	13%
TenS/1-19	16%	0%	100%	42%	5%	11%
DMBS/1-19	11%	5%	42%	100%	16%	26%
H.pylori/1-21 FabG	5%	0%	5%	14%	100%	33%
E./1-21 coli FabG	19%	10%	10%	24%	33%	100%

Legend: 10% 25% 50% 70% 90%

Figure S4 | Sequence alignment and similarity matrix of the substrate binding region. Aligned is the region which builds the substrate binding helices in TenS, DmbS and FabG as well as their counterparts in EcPKS and mFAS.The Alignment was created with Unipro UGENE using the Clustl Algorithm.

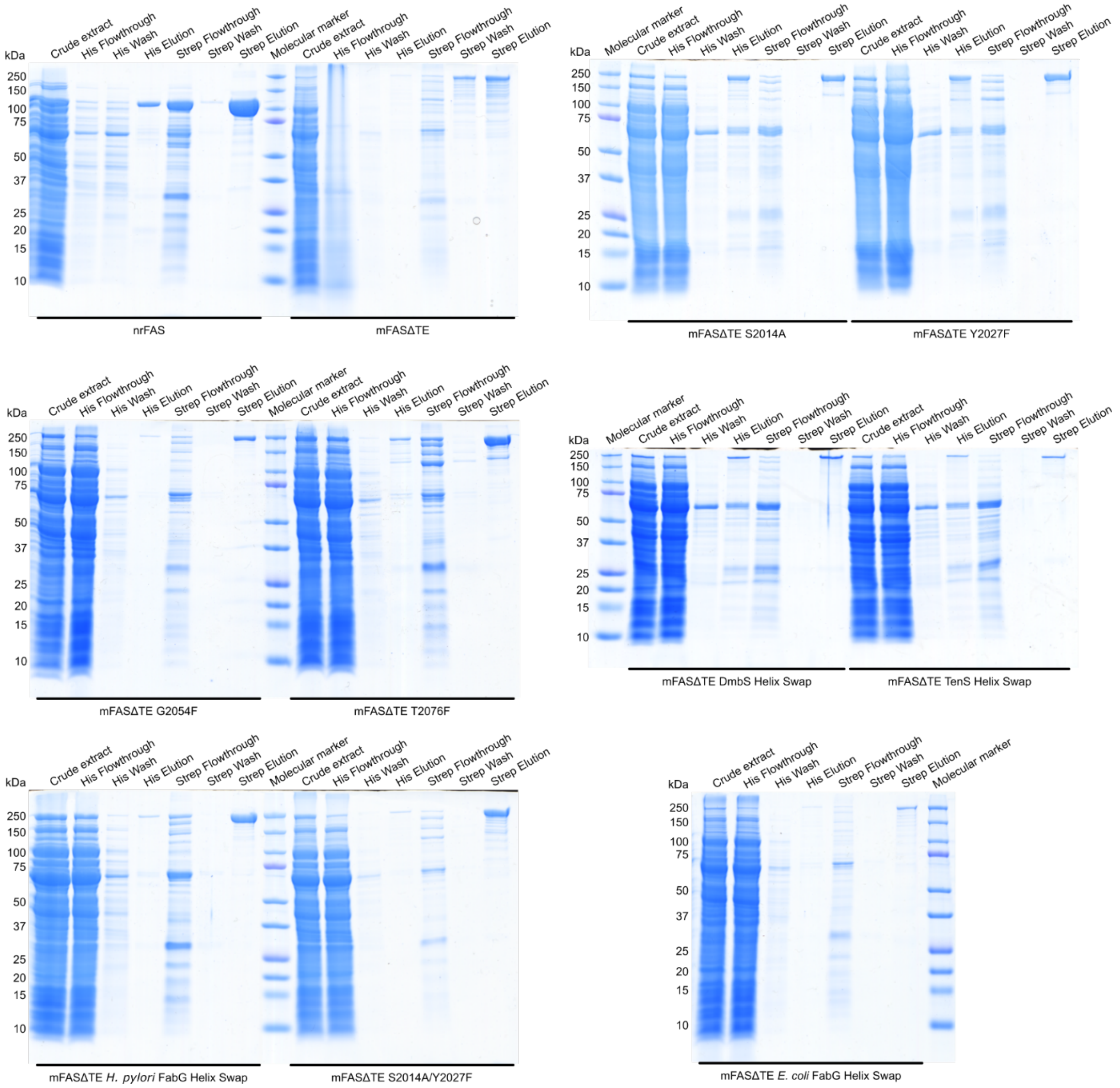

Figure S5 | Representative SDS-PAGE analysis of the protein purification procedure. The evident molecular weights of mFAS mutants recombinantly produced in *E. coli* align with the calculated molecular weights of 243–244 kDa and 108 kDa of the mFASΔTE and the nrFAS respectively.

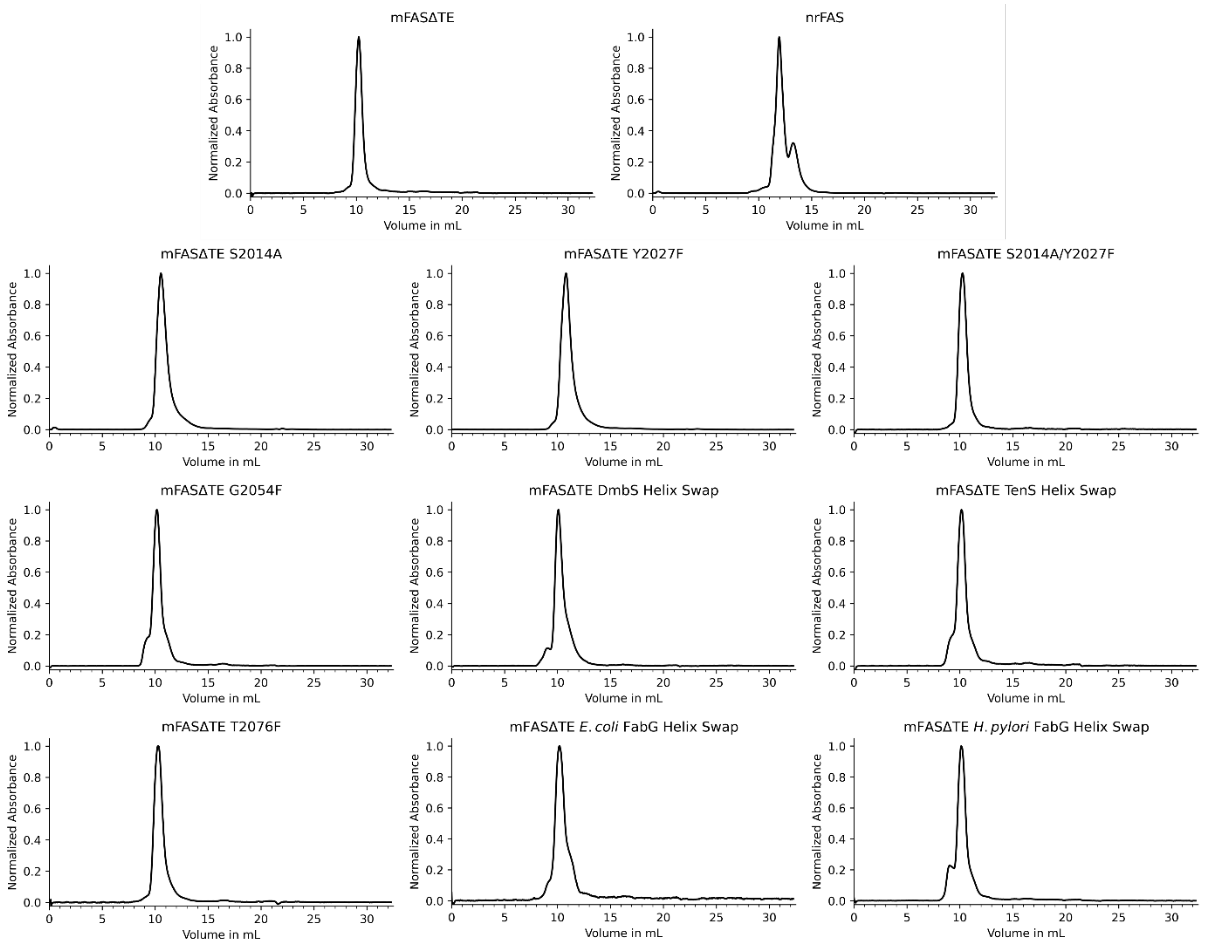

Figure S6 | Size exclusion chromatogram of all constructs. The shown normalized chromatograms are representative of all mFAS mutants produced. All mFASΔTE variants show a pronounced peak at 10 to 11 mL elution volume corresponding to the dimeric state of the construct. A following shoulder indicates remaining monomeric protein. A preceding peak/shoulder at 9 mL elution volume points to aggregate. The chromatogram of the nrFAS exhibits the analogues characteristics with a shift +2 mL elution volume. Fractions of the dimeric state were concentrated and used for further experiments.

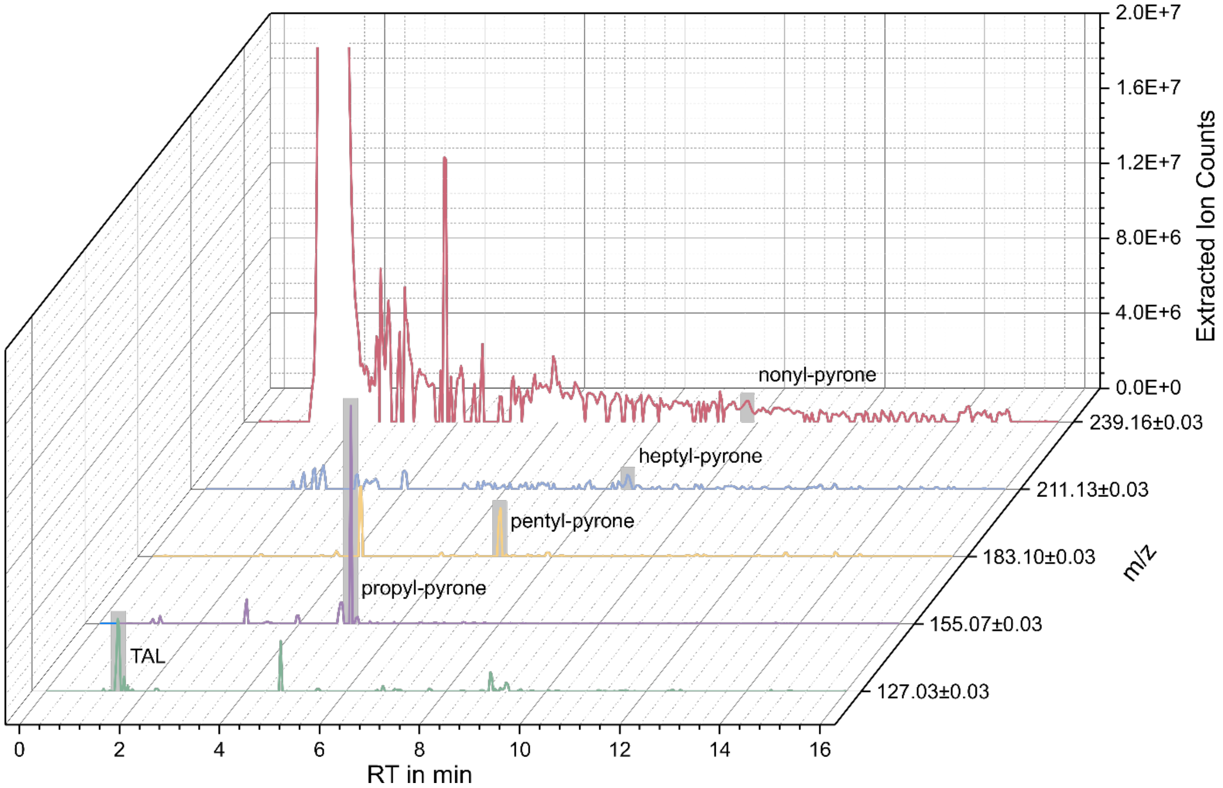

Figure S7 | Representative extracted ion chromatograms of the via LC-MS analyzed product species. The chromatograms depicted originate from one technical sample of the mFASΔTE *E. coli* FabG Helix Swap mutant. Extracted are the chromatograms for the protonated species of the expected products with an m/z tolerance of 0.03 (TAL: 127.03, propyl-pyrone: 155.07, pentyl-pyrone: 183.10, heptyl-pyrone: 211.13, nonyl-pyrone: 239.16). Peaks targeted for the automated integration are highlighted using grey boxes.

Sequence Information

Table S3 | Primers used in this work. The fragments for the In-Fusion Cloning containing mutations in the mFAS KR were created using the following primers. Template for all constructs was pAR88 (mFASΔTE).

Target Mutation	Primer Name	Primer Sequence 5'-3'
S2014A	PrDL122	CTACTTTGTGGCCTTCTCCGCAGTAAGCTGCGGGCGTGGTAATG
	PrDL123	CATTACCACGCCCGCAGCTTACTGCGGAGAAGGCCACAAAGTAG
Y2027F	PrDL124	GTAATGCTGGCCAAACTAACTTCGGCTTCGCCAACTCTACCATG
	PrDL125	CATGGTAGAGTTGGCGAAGCCGAAGTTAGTTTGGCCAGCATTAC
G2054F	PrDL126	CCTTGCCGTGCAGTGGTTTGCCATTGGTGACGTGGGC
	PrDL127	GCCCACGTCACCAATGGCAAACCACTGCACGGCAAGG
T2076F	PrDL132	CAATGACACAGTCATCGGAGGTTTCCTGCCTCAGCGCATCTCCTC
	PrDL133	GAGGAGATGCGCTGAGGCAGGAAACCTCCGATGACTGTGTCATTG
DmbS Helix Swap	PrDL120	GCAACATAGCTACCATGCGAGCTATGAGGCTCTCTATCTCCTCCTGCATGGAG
	PrDL121	TGGTAGCTATGTTGCTCTGTATTCTGTTGTCGTCGACTTCCAGGACAATGCCCAC
TenS Helix Swap	PrDL118	TGAGCCTAGGTACCACGCGAGTCATGAGTGTCTCTATCTCCTCCTGCATGGAG
	PrDL119	TGGTACCTAGGCTCATCTGCACCTTGGTGTCGTCAACTTCCAGGACAATGCCCAC
E. coli FabG Helix Swap	PrDL182	GTATCCTGGCGCAGGTTCCTGCGGGTCGCCTCGGCGGCATCTCCTCCTGCATGGAG
	PrDL183	CCTGCGCCAGGATACCCGCACGCTGGTCATCGCTCAGCGCTTCCAGGACAATGCCCAC
H. pylori FabG Helix Swap	PrDL186	ATTATGTTAAAAACATTCCTTTAAACAGGCTAGGGTCTATCTCCTCCTGCATGGAG
	PrDL187	TGTTTTTAACATAATCCGCTTTGAGTTCGTCTTTCAAATTTTCCAGGACAATGCCCAC

Table S4 | Sequence information of the constructs used in this work. The amino acid and DNA sequences of the constructs pAR88 (mFASΔTE) and pAR127 (nrFAS), as well as for the swapped helices of DmbS, TenS, the *E. coli* FabG, and the *H. pylori* FabG are listed below. The mutated positions are highlighted.

Construct / Helix	Sequence from start to stop codon / as swapped
pAR88 (mFASΔTE, Strep- and His-tagged)<br>Positions 2014 (green), 2027 (cyan), 2054 (magenta), and 2076 (red), as well as the helix swap sequence (yellow) are highlighted	MSAWSHPQFEKGGGSGGGSGGSAWSHPQFEKGAGSEEVVIAGMSGKLPESENLQEFWANLIGGVDMVTDD<br>DRRWKAGLYGLPKRSGKLKDLSKFDASFFGVHPKQAHTMDPQLRLLLEVSYEAIVDGGINPASLRGTNTGV<br>WVGVSGSEASEALSRDPETLLGYSMVGCQRAMMANRLSFFFDFKGPSIALDTACSSSLLALQNAYQAIRSGEC<br>PAALVGGINLLLKPNTSVQFMKLGMLSPDGTCRSFDDSGSGYCRSEAVVAVLLTKKSLARRVYATILNAGTN<br>TDGSKEQGVTFPSGEVQEQLICSLYQPAGLAPESLEYIEAHGTGTKVGDPQELNGITRSLCAFRQAPLLIGSTK<br>SNMGHPEPASGLAALTKVLLSLEHGVWAPNLHFHNPNPEIPALLDGRLQVVDRPLPVRGGNVGINSFGFGGS<br>NVHVILQPNTRQAPAPTAHAALPHLLHASGRTLEAVQDLLEQGRQHSQDLAFVSMLNDIAATPTAAMPFRG<br>YTVLGVEGRVQEVQQVSTNKRPLWFICSGMGTQWRGMGLSLMRLDSFRESILRSDEAVKPLGVKVSDLLLST<br>DERTFDDIVHAFVSLTAIQIALIDLLTSVGLKPDGIIGHSLGEVACGYADGCLSQREAVLAAYWRGQCIKDAHL<br>PPGSMAAVGLSWEECKQRCPAGVVPACHNSEDTVTISGPQAAVNEFVEQLKQEGVFAKEVRTGGLAFHSYF<br>MEGIAPTLLQALKKVIREPRPRSARWLSTSIPEAQWQSSLARTSSAEYNVNNLVSPVLFQEALWHIPEHAVV<br>LEIAPHALLQAVLKRGVKSSCTIIPLMKRDHKDNLEFFLTNLGKVHLTGINVNPNALFPPVEFPAPRGTPLISP<br>HIKWDHSQTWDVPVAEDFPNGSSSSSATVYSIDASPESPDHYLVDHCIDGRVIFPGTGYLCLVWKTLARSLGL<br>SLEETPVVFENVSFHQATILPKTGTVALEVRLLEASHAFEVSDTGNLIVSGKVYLWEDPNSKLFDHPEVPTPP<br>ESASVSRLTQGEVYKELRLRGYDYGPQFQGICEATLEGEQGKLLWKDNWVTFMDTMLQVSILGSSQQSLQLP<br>TRVTAIYIDPATHRQKVYRLKEDTQVADVTTSRCLGITVSGGIHISRLQTTATSRRQQEQLVPTLEKFVFTPH<br>MEAECLSESTALQKELQLCKGLARALQTKATQQGLKAAMLGQEDPPQHGLPRLLAAACQLQLNGNLQLELG<br>EALAQERLLLPEDPLISGLLNSQALKACVDTALENLSTLKMKVAEVLAGEGHLYSRIPALLNTQPMLQLEYTA<br>TDRHPQALKDVQTKLQQHDVAQGQWNPSDPAPSSLGALDLLVCNCALATLGDPALALDNMVAALKEGGFL<br>LVHTVLKGHALGETLACLPSEVQPAPSLLSQEEWESLFSRKALHLVGLKRSFYGTALFLCRRAIPQEKPIFLSV<br>EDTSFQWVDSLKSTLATSSSQPVWLTAMDCPTSGVVGLVNCLRKEPGGHRIRCILLSNLSNTSHAPKLDPGS<br>PELQQVLKHDLVMNVYRDGAWGAFRHFQLEQDKPKEQTAHAFVNVLTRGDLASIRWVSSPLKHTQPSSSG<br>AQLCTVYYASLNFRDIMLATGKLSPDAIPGKWASRDCMLGMEFSGRDRCGRRVMGLVPAEGLATSVLLSSDF<br>LWDVPSSWTLEEAASVPVVYTTAYYSLVVRGRIQRGETVLIHSGSGGVGQAAISIALSLGCRVFTTVGSAEKR<br>AYLQARFPQLDDTSFANSRDTSFEQHVLLHTGGKGVDLVLNSLAEEKLQASVRCLAQHGRFLEIGKFDLSNN<br>HPLGMAIFLKNVTFHGILLDALFEEANDSWREVAALLKAGIRDGVVKPLKCTVFPKAQVEDAFRYMAQGKH<br>IGKVLVQVREEEPEAVLPGAQPTLISAISKTFCPAHKSYIITGGLGGFGLELARWLVLRGAQRLVLTSRSGIRTG<br>YQAKHIREWRRQGIQVLVSTSNVSSLEGARALIAEATKLGPVGGVFNLAMVLRDAMLENQTPELFQDVNKP<br>KYNGTLNLDRATREACPELDYFVAFSSVSCGRGNAGQTNYGFANSTMERICEQRRHDGLPGLAVQWGAIGD<br>VGIVLEAMGTNDTVIGGTLPQRISSCMEVLDLFLNQPHAVLSSFVLAEKKAVAHGDGDTQRDLVKAVAHILGI<br>RDLAGINLDSTLADLGLDSLMGVEVRQILEREHDLVLPMREVRQLTLRKLQEMSSKTDSATDTTLEHHHHH<br>HHH

Construct / Helix	Sequence from start to stop codon / as swapped
pAR88 (mFASΔTE, Strep- and His-tagged)<br>Positions 2014 (green), 2027 (cyan), 2054 (magenta), and 2076 (red), as well as the helix swap sequence (yellow) are highlighted	ATGAGCGCTTGGAGCCATCCACAATTTGAGAAGGGTGGAGGTTCTGGCGGTGGATCGGGAGGTTCAGCGTG<br>GAGCCACCCGCAGTTCGAAAAAGGCGCCGGATCCGAGGAGGTGGTGATAGCCGGTATGTCGGGGAAGTTGC<br>CCGAGTCAGAGAACCTACAGGAGTTCTGGGCCAACCTCATTGGTGGTGTGGACATGGTCACAGATGATGAC<br>AGGAGATGGAAGGCTGGGCTCTATGGATTACCCAAGCGGTCTGGAAAGCTGAAGGATCTCTCCAAGTTCGA<br>CGCCTCCTTTTTTGGGGTCCACCCCAAGCAGGCACACACAATGGACCCCCAGCTTCGGCTGCTGTTGGAAGT<br>CAGCTATGAAGCAATTGTGGATGGAGGTATCAACCCAGCCTCACTCCGAGGAACGAACACTGGCGTCTGGG<br>TGGGTGTGAGTGGTTCAGAGGCATCCGAGGCCCTTAGCAGAGATCCCGAGACGCTTCTGGGCTACAGCATG<br>GTGGGCTGCCAGCGTGCAATGATGGCCAACCGGCTCTCTTTCTTCTTCGACTTCAAAGGACCAAGCATTGCC<br>CTGGACACAGCCTGCTCCTCCAGCTTGCTGGCACTACAGAATGCCTACCAGGCCATCCGTAGTGGGGAATGC<br>CCCGCGGCCCTTGTGGGTGGGATCAACCTGCTCCTGAAGCCGAACACCTCTGTGCAGTTCATGAAGCTGGGC<br>ATGCTCAGCCCGGACGGCACCTGCAGATCCTTTGATGATTCAGGGAGTGGATATTGTCGCTCTGAGGCTGT<br>TGTAGCAGTTCTGCTGACTAAGAAGTCCCTGGCTCGGCGGGTCTATGCCACGATTCTGAATGCCGGCACCAA<br>TACAGATGGCAGCAAGGAGCAAGGTGTAACATTCCCCTCTGGAGAAGTCCAAGAACAACTCATCTGCTCTC<br>TGTATCAGCCAGCTGGTCTGGCCCCGGAGTCGCTTGAGTATATTGAAGCCCATGGCACGGGCACCAAGGTG<br>GGTGACCCCCAGGAACTGAATGGCATTACTCGGTCCCTGTGCGCCTTCCGCCAGGCCCCTCTGTTAATTGGC<br>TCCACCAAATCCAACATGGGACACCCTGAGCCTGCCTCTGGGCTTGCAGCCCTGACCAAGGTGCTGTTATCC<br>CTGGAGCATGGGGTCTGGGCCCCTAACCTGCACTTCCACAACCCCAACCCTGAGATCCCAGCACTTCTTGAT<br>GGGCGGCTGCAGGTGGTCGATAGGCCCCTGCCTGTTCGTGGTGGCAACGTGGGCATCAACTCATTTGGCTTC<br>GGAGGCTCCAATGTTCATGTCATCCTCCAGCCCAACACACGGCAGGCCCCTGCGCCCACTGCACACGCTGCC<br>CTTCCCCATTTGCTGCACGCCAGTGGACGCACCTTAGAGGCAGTGCAGGACCTGCTGGAACAGGGCCGCCAG<br>CACAGCCAGGACCTGGCCTTTGTGAGCATGCTCAATGACATTGCGGCAACCCCTACAGCAGCCATGCCCTTC<br>AGGGGTTACACTGTGCTAGGTGTTGAGGGCCGTGTCCAAGAAGTGCAGCAAGTGTCCACCAACAAGCGCCC<br>ACTCTGGTTCATCTGCTCAGGGATGGGCACGCAGTGGCGCGGGATGGGGCTGAGCCTCATGCGCCTGGACA<br>GCTTCCGTGAGTCTATCCTGCGCTCCGATGAGGCTGTGAAGCCGTTGGGAGTGAAAGTGTCAGATCTGCTG<br>TTGAGCACAGATGAGCGCACCTTTGATGACATCGTGCATGCCTTTGTGAGCCTCACTGCCATCCAGATTGCC<br>CTCATCGACCTACTGACTTCTGTGGGACTGAAACCTGACGGCATCATTGGGCACTCCTTGGGAGAGGTTGCC<br>TGTGGCTATGCAGATGGCTGTCTCTCCCAGAGAGAGGCTGTGCTTGCAGCTTACTGGCGAGGCCAGTGCAT<br>CAAAGATGCCCACCTCCCGCCTGGATCCATGGCAGCTGTTGGTTTGTCCTGGGAGGAATGTAAACAGCGCTG<br>CCCCGCTGGCGTGGTGCCTGCCTGCCACAACTCTGAGGACACCGTGACCATCTCTGGACCTCAGGCTGCAGT<br>GAATGAATTTGTGGAGCAGCTAAAGCAAGAAGGTGTGTTTGCCAAGGAGGTACGAACAGGAGGCCTGGCTT<br>TCCACTCCTACTTCATGGAAGGAATTGCCCCCACATTGCTGCAGGCTCTCAAGAAGGTGATCCGGGAACCAC<br>GGCCGCGCTCGGCTCGATGGCTCAGCACCTCTATCCCTGAGGCCCAGTGGCAGAGCAGCCTGGCCCGCACAT<br>CTTCTGCCGAGTACAATGTCAACAACCTGGTGAGCCCTGTGCTCTTCCAGGAAGCACTGTGGCACATCCCTG<br>AGCATGCCGTGGTGCTGGAGATTGCGCCCCACGCACTGTTGCAGGCTGTCCTGAAGCGAGGCGTGAAGTCC<br>AGCTGCACCATCATTCCCTTGATGAAGAGGGATCATAAAGATAACTTGGAGTTCTTTCTCACCAACCTTGG<br>CAAGGTGCACCTCACAGGCATCAATGTCAACCCTAACGCCTTGTTCCCACCTGTGGAGTTCCCGGCTCCCCG<br>AGGGACTCCTCTCATCTCCCCTCACATCAAGTGGGACCACAGTCAGACTTGGGATGTCCCGGTTGCTGAGGA<br>CTTCCCAAACGGCTCCAGCTCCTCCTCTGCTACAGTCTACAGCATCGACGCCAGTCCTGAGTCGCCCGACCA<br>CTACCTGGTAGACCACTGCATTGACGGCCGGGTCATCTTCCCTGGCACTGGCTACCTGTGCCTGGTGTGGAA<br>GACACTGGCTCGCAGCCTGGGCTTGTCCCTAGAAGAGACCCCTGTGGTATTTGAGAATGTGTCGTTTCATC<br>AGGCCACTATACTACCCAAGACAGGAACCGTGGCGCTGGAGGTGAGGCTGCTAGAGGCCTCCCATGCCTTT<br>GAGGTGTCTGACACTGGCAATCTGATTGTGAGCGGAAAAGTGTACCTGTGGGAAGACCCGAACTCCAAGTT<br>ATTCGACCACCCAGAAGTCCCAACACCCCCTGAGTCTGCATCGGTCTCCCGCCTGACCCAGGGAGAAGTATA<br>CAAGGAGCTGCGGCTGCGTGGCTATGATTATGGCCCTCAGTTCCAGGGCATCTGTGAGGCCACCCTTGAAG<br>GTGAACAAGGCAAGCTGCTCTGGAAAGATAACTGGGTGACCTTCATGGACACAATGCTGCAGGTATCCATT<br>CTGGGTTCTAGCCAGCAGAGTCTACAGCTACCTACCCGTGTGACCGCCATCTATATCGACCCTGCCACCCAC<br>CGTCAGAAGGTGTACAGGCTGAAGGAGGACACTCAAGTGGCTGATGTGACAACGAGCCGCTGTCTGGGCAT<br>AACGGTCTCTGGTGGTATCCACATCTCAAGACTACAGACGACAGCAACCTCACGGCGGCAGCAAGAACAGC<br>TGGTCCCCACCTTGGAAAAGTTCGTTTTCACACCGCACATGGAGGCTGAGTGCCTGTCTGAGAGCACTGCCC<br>TGCAGAAGGAGCTGCAACTGTGCAAGGGTCTGGCACGGGCTCTGCAGACCAAGGCCACCCAGCAAGGGCTG<br>AAGGCGGCAATGCTTGGGCAAGAGGACCCTCCACAGCACGGGCTGCCTCGACTCCTGGCAGCTGCTTGCCAG<br>TTGCAGCTCAACGGGAACCTGCAGCTGGAGCTGGGAGAAGCGCTGGCTCAAGAGAGGCTCCTGCTGCCAGA<br>AGACCCTCTGATCAGTGGCCTCCTCAACTCCCAGGCCCTCAAGGCCTGCGTAGACACAGCCCTGGAGAACTT<br>GTCTACTCTCAAGATGAAGGTGGCAGAGGTGCTGGCTGGAGAAGGCCACTTGTATTCCCGAATCCCGGCAC<br>TGCTCAACACCCAGCCCATGCTACAACTGGAATACACAGCCACCGACCGGCACCCCCAGGCCCTGAAGGATG<br>TTCAGACCAAACTGCAGCAGCATGATGTGGCGCAGGGCCAGTGGAACCCTTCCGACCCTGCGCCCAGCAGCC<br>TGGGTGCCCTTGACCTTCTGGTGTGCAACTGTGCATTAGCCACCCTGGGGGATCCAGCCTTGGCCCTGGACA<br>ACATGGTAGCTGCCCTCAAGGAAGGTGGTTTCCTGCTAGTGCACACAGTGCTCAAAGGACATGCCCTTGGG<br>GAGACCCTGGCCTGCCTACCCTCTGAGGTGCAGCCTGCGCCCAGCCTCCTAAGCCAGGAGGAGTGGGAGAGC<br>CTGTTCTCGAGGAAGGCACTACACCTGGTGGGCCTTAAAAGGTCCTTCTACGGTACTGCGCTGTTCCTGTGC<br>CGGCGAGCCATCCCACAGGAGAAACCTATCTTCCTGTCTGTGGAGGATACCAGCTTCCAGTGGGTGGACTCT<br>CTGAAGAGCACTCTGGCCACGTCCTCCTCCCAGCCTGTGTGGCTAACGGCCATGGACTGCCCCACCTCGGGT<br>GTGGTGGGTTTGGTGAATTGTCTCCGAAAAGAGCCGGGTGGACACCGGATTCGGTGTATCCTGCTGTCCAA<br>CCTCAGCAACACATCTCACGCCCCCAAGTTGGACCCTGGCTCTCCAGAGCTACAGCAGGTGCTAAAGCATGA<br>CCTCGTGATGAACGTGTACCGGGACGGGGCCTGGGGTGCCTTCCGTCACTTCCAGTTAGAGCAGGACAAGCC<br>CAAGGAGCAGACAGCGCATGCCTTTGTAAACGTCCTCACCCGAGGGGACCTCGCCTCCATCCGCTGGGTCTC<br>CTCCCCCCTGAAGCACACGCAGCCCTCGAGCTCAGGAGCACAGCTCTGCACTGTCTACTACGCCTCACTGAA<br>CTTCCGAGACATCATGCTGGCCACGGGCAAGCTGTCCCCTGATGCCATTCCAGGTAAATGGGCCAGCCGAGA<br>CTGCATGCTCGGCATGGAGTTCTCAGGCCGGGATAGGTGTGGCCGGCGTGTGATGGGGCTGGTTCCTGCAG<br>AAGGCCTGGCCACCTCAGTCCTGCTATCATCTGACTTCCTCTGGGATGTACCCTCCAGCTGGACCCTGGAGG<br>AGGCGGCCTCTGTGCCCGTCGTCTATACCACTGCTTACTACTCGTTAGTGGTTCGCGGGCGCATCCAGCGTG<br>GGGAGACCGTGCTCATCCACTCAGGTTCAGGTGGTGTGGGCCAAGCGGCCATTTCCATTGCCCTCAGTCTGG<br>GCTGCCGCGTCTTCACCACTGTGGGCTCTGCAGAGAAGCGAGCATACCTCCAGGCCAGGTTCCCTCAGCTTG<br>ATGACACCAGCTTTGCCAACTCGAGGGACACATCATTTGAGCAGCACGTGTTACTGCACACAGGTGGCAAA

Construct / Helix	Sequence from start to stop codon / as swapped
	GGGGTCGACCTGGTCCTCAACTCACTGGCAGAAGAGAAGCTGCAGGCCAGTGTGCGGTGCTTGGCTCAGCA<br>TGGTCGCTTCTTAGAGATTGGCAAATTTGATCTTTCTAACAACCACCCTCTGGGCATGGCTATCTTCTTGAA<br>GAACGTCACTTTCCATGGGATCCTGCTGGACGCCCTTTTTGAGGAGGCCAATGACAGCTGGCGGGAGGTGG<br>CGGCACTCCTGAAGGCTGGCATTCGTGATGGAGTCGTGAAGCCCCTCAAGTGCACAGTGTTTCCCAAGGCCC<br>AGGTGGAAGATGCCTTCCGCTACATGGCTCAGGGGAAACACATTGGCAAAGTCCTTGTCCAGGTACGGGAG<br>GAGGAGCCTGAGGCTGTGCTGCCAGGGGCTCAGCCCACCCTGATTTCTGCCATCTCCAAGACCTTCTGCCCA<br>GCCCATAAGAGTTACATCATCACTGGTGGCCTAGGTGGCTTTGGCCTGGAGCTGGCCCGGTGGCTCGTGCTT<br>CGCGGAGCCCAGAGGCTTGTGCTGACTTCCCGATCTGGAATCCGCACCGGCTACCAAGCCAAGCACATTCGG<br>GAGTGGAGACGCCAGGGCATCCAAGTGCTCGTGTCAACAAGCAACGTGAGCTCACTGGAGGGGGCCCGTGC<br>TCTCATCGCCGAAGCCACAAAGCTGGGGCCCGTTGGGGGTGTCTTCAACCTGGCCATGGTTTTGAGGGATGC<br>CATGCTGGAGAACCAGACCCCAGAGCTCTTCCAGGATGTCAACAAGCCCAAATACAATGGCACCCTGAACCT<br>TGACAGGGCAACCCGGGAAGCCTGCCCTGAGCTGGACTACTTTGTGGCCTTCTCCTCTGTAAGCTGCGGGCG<br>TGGTAATGCTGGCCAAACTAACTACGGCTTCGCCAACTCTACCATGGAGCGTATATGTGAACAGCGCAGGC<br>ACGATGGCCTCCCAGGCCTTGCCGTGCAGTGGGGTGCCATTGGTGACGTGGGCATTGTCCTGGAAGCGATG<br>GGCACCAATGACACAGTCATCGGAGGTACGCTGCCTCAGCGCATCTCCTCCTGCATGGAGGTACTGGACCTC<br>TTCCTGAATCAGCCCCACGCAGTCCTGAGCAGCTTTGTGCTGGCAGAGAAGAAAGCTGTGGCCCATGGGGA<br>CGGGGACACCCAGAGGGATCTGGTGAAAGCTGTAGCACACATCCTAGGCATCCGAGACCTCGCAGGTATTA<br>ACCTGGACAGCACGCTGGCAGACCTCGGCCTGGACTCGCTCATGGGTGTGGAAGTTCGTCAGATCCTGGAAC<br>GAGAACACGATCTGGTGCTGCCCATGCGTGAGGTGCGGCAGCTCACGCTGCGGAAACTTCAGGAAATGTCC<br>TCCAAGACTGACTCGGCTACTGACACGACACTCGAGCATCATCACCACCACCACCACCAC
pAR127 (nrFAS, Strep- and His-tagged)	MSAWSHPQFEKGGGSGGGSGGSAWSHPQFEKGAGSEEVVIAGMSGKLPESENLQEFWANLIGGVDMVTDD<br>DRRWKAGLYGLPKRSGKLKDLSKFDASFFGVHPKQAHTMDPQLRLLLEVSYEAIVDGGINPASLRGTNTGV<br>WVGVSGSEASEALSRDPETLLGYSMVGCQRAMMANRLSFFFDFKGPSIALDTACSSSLLALQNAYQAIRSGEC<br>PAALVGGINLLLKPNTSVQFMKLGMLSPDGTCRSFDDSGSGYCRSEAVVAVLLTKKSLARRVYATILNAGTN<br>TDGSKEQGVTFPSGEVQEQLICSLYQPAGLAPESLEYIEAHGTGTKVGDPQELNGITRSLCAFRQAPLLIGSTK<br>SNMGHPEPASGLAALTKVLLSLEHGVWAPNLHFHNPNPEIPALLDGRLQVVDRPLPVRGGNVGINSFGFGGS<br>NVHVILQPNTRQAPAPTAHAALPHLLHASGRTLEAVQDLLEQGRQHSQDLAFVSMLNDIAATPTAAMPFRG<br>YTVLGVEGRVQEVQQVSTNKRPLWFICSGMGTQWRGMGLSLMRLDSFRESILRSDEAVKPLGVKVSDLLLST<br>DERTFDDIVHAFVSLTAIQIALIDLLTSVGLKPDGIIGHSLGEVACGYADGCLSQREAVLAAYWRGQCIKDAHL<br>PPGSMAAVGLSWEECKQRCPAGVVPACHNSEDTVTISGPQAAVNEFVEQLKQEGVFAKEVRTGGLAFHSYF<br>MEGIAPTLLQALKKVIREPRPRSARWLSTSIPEAQWQSSLARTSSAEYNVNNLVSPVLFQEALWHIPEHAVV<br>LEIAPHALLQAVLKRGVKSSCTIIPLMKRDHKDNLEFFLTNLGKVHLTGINVNPNALFPPVEFPAPRGTPLISP<br>HIKWDHSQTWDVPVAEDFPNGSSSSSATVYSIDASAEKKAVAHGDGDTQRDLVKAVAHILGIRDLAGINLDS<br>TLADLGLDSLMGVEVRQILEREHDLVLPMREVRQLTLRKLQEMSSKTDSATDTTLEHHHHHHHH
pAR127 (nrFAS, Strep- and His-tagged)	ATGAGCGCTTGGAGCCATCCACAATTTGAGAAGGGTGGAGGTTCTGGCGGTGGATCGGGAGGTTCAGCGTG<br>GAGCCACCCGCAGTTCGAAAAAGGCGCCGGATCCGAGGAGGTGGTGATAGCCGGTATGTCGGGGAAGTTGC<br>CCGAGTCAGAGAACCTACAGGAGTTCTGGGCCAACCTCATTGGTGGTGTGGACATGGTCACAGATGATGAC<br>AGGAGATGGAAGGCTGGGCTCTATGGATTACCCAAGCGGTCTGGAAAGCTGAAGGATCTCTCCAAGTTCGA<br>CGCCTCCTTTTTTGGGGTCCACCCCAAGCAGGCACACACAATGGACCCCCAGCTTCGGCTGCTGTTGGAAGT<br>CAGCTATGAAGCAATTGTGGATGGAGGTATCAACCCAGCCTCACTCCGAGGAACGAACACTGGCGTCTGGG<br>TGGGTGTGAGTGGTTCAGAGGCATCCGAGGCCCTTAGCAGAGATCCCGAGACGCTTCTGGGCTACAGCATG<br>GTGGGCTGCCAGCGTGCAATGATGGCCAACCGGCTCTCTTTCTTCTTCGACTTCAAAGGACCAAGCATTGCC<br>CTGGACACAGCCTGCTCCTCCAGCTTGCTGGCACTACAGAATGCCTACCAGGCCATCCGTAGTGGGGAATGC<br>CCCGCGGCCCTTGTGGGTGGGATCAACCTGCTCCTGAAGCCGAACACCTCTGTGCAGTTCATGAAGCTGGGC<br>ATGCTCAGCCCGGACGGCACCTGCAGATCCTTTGATGATTCAGGGAGTGGATATTGTCGCTCTGAGGCTGT<br>TGTAGCAGTTCTGCTGACTAAGAAGTCCCTGGCTCGGCGGGTCTATGCCACGATTCTGAATGCCGGCACCAA<br>TACAGATGGCAGCAAGGAGCAAGGTGTAACATTCCCCTCTGGAGAAGTCCAAGAACAACTCATCTGCTCTC<br>TGTATCAGCCAGCTGGTCTGGCCCCGGAGTCGCTTGAGTATATTGAAGCCCATGGCACGGGCACCAAGGTG<br>GGTGACCCCCAGGAACTGAATGGCATTACTCGGTCCCTGTGCGCCTTCCGCCAGGCCCCTCTGTTAATTGGC<br>TCCACCAAATCCAACATGGGACACCCTGAGCCTGCCTCTGGGCTTGCAGCCCTGACCAAGGTGCTGTTATCC<br>CTGGAGCATGGGGTCTGGGCCCCTAACCTGCACTTCCACAACCCCAACCCTGAGATCCCAGCACTTCTTGAT<br>GGGCGGCTGCAGGTGGTCGATAGGCCCCTGCCTGTTCGTGGTGGCAACGTGGGCATCAACTCATTTGGCTTC<br>GGAGGCTCCAATGTTCATGTCATCCTCCAGCCCAACACACGGCAGGCCCCTGCGCCCACTGCACACGCTGCC<br>CTTCCCCATTTGCTGCACGCCAGTGGACGCACCTTAGAGGCAGTGCAGGACCTGCTGGAACAGGGCCGCCAG<br>CACAGCCAGGACCTGGCCTTTGTGAGCATGCTCAATGACATTGCGGCAACCCCTACAGCAGCCATGCCCTTC<br>AGGGGTTACACTGTGCTAGGTGTTGAGGGCCGTGTCCAAGAAGTGCAGCAAGTGTCCACCAACAAGCGCCC<br>ACTCTGGTTCATCTGCTCAGGGATGGGCACGCAGTGGCGCGGGATGGGGCTGAGCCTCATGCGCCTGGACA<br>GCTTCCGTGAGTCTATCCTGCGCTCCGATGAGGCTGTGAAGCCGTTGGGAGTGAAAGTGTCAGATCTGCTG<br>TTGAGCACAGATGAGCGCACCTTTGATGACATCGTGCATGCCTTTGTGAGCCTCACTGCCATCCAGATTGCC<br>CTCATCGACCTACTGACTTCTGTGGGACTGAAACCTGACGGCATCATTGGGCACTCCTTGGGAGAGGTTGCC<br>TGTGGCTATGCAGATGGCTGTCTCTCCCAGAGAGAGGCTGTGCTTGCAGCTTACTGGCGAGGCCAGTGCAT<br>CAAAGATGCCCACCTCCCGCCTGGATCCATGGCAGCTGTTGGTTTGTCCTGGGAGGAATGTAAACAGCGCTG<br>CCCCGCTGGCGTGGTGCCTGCCTGCCACAACTCTGAGGACACCGTGACCATCTCTGGACCTCAGGCTGCAGT<br>GAATGAATTTGTGGAGCAGCTAAAGCAAGAAGGTGTGTTTGCCAAGGAGGTACGAACAGGAGGCCTGGCTT<br>TCCACTCCTACTTCATGGAAGGAATTGCCCCCACATTGCTGCAGGCTCTCAAGAAGGTGATCCGGGAACCAC<br>GGCCGCGCTCGGCTCGATGGCTCAGCACCTCTATCCCTGAGGCCCAGTGGCAGAGCAGCCTGGCCCGCACAT<br>CTTCTGCCGAGTACAATGTCAACAACCTGGTGAGCCCTGTGCTCTTCCAGGAAGCACTGTGGCACATCCCTG<br>AGCATGCCGTGGTGCTGGAGATTGCGCCCCACGCACTGTTGCAGGCTGTCCTGAAGCGAGGCGTGAAGTCC<br>AGCTGCACCATCATTCCCTTGATGAAGAGGGATCATAAAGATAACTTGGAGTTCTTTCTCACCAACCTTGG<br>CAAGGTGCACCTCACAGGCATCAATGTCAACCCTAACGCCTTGTTCCCACCTGTGGAGTTCCCGGCTCCCCG<br>AGGGACTCCTCTCATCTCCCCTCACATCAAGTGGGACCACAGTCAGACTTGGGATGTCCCGGTTGCTGAGGA<br>CTTCCCAAACGGCTCCAGCTCCTCCTCTGCTACAGTCTACAGCATCGACGCCAGTGCAGAGAAGAAAGCTGT<br>GGCCCATGGGGACGGGGACACCCAGAGGGATCTGGTGAAAGCTGTAGCACACATCCTAGGCATCCGAGACC<br>TCGCAGGTATTAACCTGGACAGCACGCTGGCAGACCTCGGCCTGGACTCGCTCATGGGTGTGGAAGTTCGTC

Construct / Helix	Sequence from start to stop codon / as swapped
	AGATCCTGGAACGAGAACACGATCTGGTGCTGCCCATGCGTGAGGTGCGGCAGCTCACGCTGCGGAAACTT CAGGAAATGTCCTCCAAGACTGACTCGGCTACTGACACGACACTCGAGCATCATCACCACCACCACCACCAC
DmbS Helix	VDDNRIQSNIATMRAMRLS
	GTCGACGACAACAGAATACAGAGCAACATAGCTACCATGCGAGCTATGAGGCTCTCT
TenS Helix	VDDTKVQMSLGTTRVMSVS
	GTTGACGACACCAAGGTGCAGATGAGCCTAGGTACCACGCGAGTCATGAGTGTCTCT
E. coli FabG Helix	ALSDDQRAGILAQVPAGRLGG
	GCGCTGAGCGATGACCAGCGTGCGGGTATCCTGGCGCAGGTTCCTGCGGGTCGCCTCGGCGGC
H. pylori FabG Helix	NLKDELKADYVKNIPLNRLGS
	AATTTGAAAGACGAACTCAAAGCGGATTATGTTAAAAACATTCCTTTAAACAGGCTAGGGTCT
